# Understanding the molecular mechanisms of human microtia via a pig model of *HOXA1* syndrome

**DOI:** 10.1242/dmm.018291

**Published:** 2015-06-01

**Authors:** Ruimin Qiao, Yuyong He, Bo Pan, Shijun Xiao, Xufei Zhang, Jing Li, Zhiyan Zhang, Yuan Hong, Yuyun Xing, Jun Ren

**Affiliations:** ^1^ Key Laboratory for Animal Biotechnology of Jiangxi Province and the Ministry of Agriculture of China, Jiangxi Agricultural University, Nanchang 330045, People's Republic of China; ^2^ College of Animal Science and Veterinary Medicine, Henan Agricultural University, Zhengzhou 450002, People's Republic of China; ^3^ Plastic Surgery Hospital, Peking Union Medical College, Beijing 100144, People's Republic of China

**Keywords:** Microtia, Pig model, Molecular mechanism, *HOXA1*, *EVC2*

## Abstract

Microtia is a congenital malformation of the outer ears. Although both genetic and environmental components have been implicated in microtia, the genetic causes of this innate disorder are poorly understood. Pigs have naturally occurring diseases comparable to those in humans, providing exceptional opportunity to dissect the molecular mechanism of human inherited diseases. Here we first demonstrated that a truncating mutation in *HOXA1* causes a monogenic disorder of microtia in pigs. We further performed RNA sequencing (RNA-Seq) analysis on affected and healthy pig embryos (day 14.25). We identified a list of 337 differentially expressed genes (DEGs) between the normal and mutant samples, shedding light on the transcriptional network involving *HOXA1*. The DEGs are enriched in biological processes related to cardiovascular system and embryonic development, and neurological, renal and urological diseases. Aberrant expressions of many DEGs have been implicated in human innate deformities corresponding to microtia-associated syndromes. After applying three prioritizing algorithms, we highlighted appealing candidate genes for human microtia from the 337 DEGs. We searched for coding variants of functional significance within six candidate genes in 147 microtia-affected individuals. Of note, we identified one *EVC2* non-synonymous mutation (p.Asp1174Asn) as a potential disease-implicating variant for a human microtia-associated syndrome. The findings advance our understanding of the molecular mechanisms underlying human microtia, and provide an interesting example of the characterization of human disease-predisposing variants using pig models.

## Abstract (precis)

**Summary:** A pig model of HOXA1 syndrome provides novel insight into the molecular mechanisms of human microtia.

## INTRODUCTION

Microtia is a congenital deformity of the out ear, characterized by a small, abnormally shaped auricle. It is usually accompanied by narrowed, blocked or absent ear canal and underdeveloped middle ear because the out ear and the middle ear evolve from a common embryological origin (Kountakis et al., 1995). Microtia can occur unilaterally or bilaterally. More than 80% of microtia cases are unilateral. In the unilateral microtia, the right ear is more frequently affected, accounting for ∼60% of the unilateral cases; the unaffected ear usually has normal hearing (Castilla and Orioli, 1986; Harris et al., 1996; Jafek et al., 1975; Mastroiacovo et al., 1995). In bilateral cases, affected individuals often suffer from hearing impairment and require surgical ear reconstruction. The reported prevalence of microtia varies from 0.83 to 17.4 per 10,000 births in different ethnic populations, and the occurrence of this disorder is estimated to be higher in males, with a sex ratio of 1.5 (Canfield et al., 2009; Forrester and Merz, 2005; Harris et al., 1996; Shaw et al., 2004; Suutarla et al., 2007). The etiology of this wide variability and different gender distribution remains largely unknown.

Several grading systems have been proposed for microtia, and the most frequently used system is the Marx classification (Marx, 1926). In this classification, microtia is divided into four grades. Individuals with Grade I show all the normal ear components but have a smaller auricle. Grade II is characterized by absent normal features of the external ear. Grade III is the most common form, in which only a rudiment of soft tissue is present. The extreme form is Grade IV (anotia), which is characterized by the absence of an external ear or auditory canal.

Microtia can occur as an isolated condition, or in conjunction with other abnormalities, which mainly occurs in bilateral cases. The most common associated malformations include oral clefts, eyelid defects, facial asymmetry, renal abnormalities, cardiac defects, polydactyly and vertebral deformities (Harris et al., 1996; Mastroiacovo et al., 1995; Wang et al., 2001). These anomalies are also associated with a spectrum of syndromes, including the most common oculo-auriculo-vertebral spectrum (Tasse et al., 2005). Although the causes of microtia and its associated syndromes are poorly understood, strong evidence supports the contribution of genetic and environmental components. Various familial cases with Mendelian modes of inheritance have been reported (Alasti et al., 2008; Bicknell et al., 2011; Guernsey et al., 2011; Schaefer et al., 2014; Tekin et al., 2008; Tischfield et al., 2005). Mouse model studies have uncovered a list of genes associated with microtia, and illustrated several signaling pathways, including BMP, WNT, FGF and retinoic acid, that play a role in outer-ear development (for a review, see Luquetti et al., 2012). However, few genes and disease-causing variants that are responsible for human microtia have been unequivocally identified to date (Luquetti et al., 2012).

Naturally occurring mutations with hereditary defects in domestic animals provide an opportunity to identify further genetic causes of human congenital anomalies. Successful examples have been illustrated in a wide variety of genetic defects in, for example, dogs (Grall et al., 2012; Safra et al., 2013), cattle (Charlier et al., 2008), pigs (Milan et al., 2000) and horses (Rosengren Pielberg et al., 2008). The pig is an important biochemical model for human diseases. For example, humans have more similar physiological and anatomical features of the hearing system with pigs than with mice. Like humans, pigs can hear at birth; whereas cochlea development in mice differs from that in most mammals, including humans and pigs. The pig model is thus better than the mouse model for understanding human hearing diseases. Here, we performed a genome-wide association analysis and capture-based resequencing of the target region to identify the causative mutation for a familial case of pig microtia. Our further whole-genome RNA sequencing (RNA-Seq) analysis on affected and unaffected embryos clarified a genetic cascade of downstream genes of the responsible gene, enabling us to highlight a list of appealing candidate genes and one disease-implicating variant for human microtia. The findings provide novel insights into the molecular mechanisms underlying human microtia-associated syndromes.

**TRANSLATIONAL IMPACT**

**Clinical issue**

Microtia is a congenital abnormality (an abnormality present at birth) of the external ears. In individuals with microtia, the visible portion of the external ear (the auricle) is small and abnormally shaped on one or both sides of the head. People with the disorder usually have abnormalities in the ear canal and middle ear that lead to hearing impairment. They also often have malformations in other parts of the body, such as facial abnormalities and vertebral deformities. Both genetic and environmental components contribute to microtia, but the genetic causes of this innate condition are poorly understood. Notably, although mouse studies have identified several genes that are associated with microtia, because the development of the mouse hearing system is very different to that of the human hearing system, few genes or disease-causing genes variants for human microtia have been unequivocally identified using mouse models. However, the hearing system of the pig, unlike that of the mouse, shares many physiological and anatomical characteristics with that of humans.

**Results**

Here, the authors show that a naturally occurring truncating mutation in the *HOXA1* gene causes a monogenic (single gene) microtia disorder in pigs. They identify 337 genes that are differentially expressed by affected and healthy pigs during embryonic development, thereby shedding light on the transcriptional network that is involved in *HOXA1*-mediated development of the external ears. The authors then use three prioritization algorithms to highlight several candidates among these differentially expressed genes for human microtia. Finally, the authors identify a protein-altering mutation in *EVC2*, one of the candidate genes, that is potentially responsible for a human microtia-associated syndrome characterized by ear and rib anomalies.

**Implications and future directions**

By uncovering the relationship between *EVC2* mutations and a human microtia-associated syndrome, this study establishes a new large-animal model for the study of human microtia diseases. The identification of *EVC* and other candidate genes for microtia substantially advances our understanding of the mechanisms underlying microtia and should facilitate the characterization of additional gene variants that cause this congenital disease. Moreover, this study reveals novel therapeutic targets for human microtia and, more generally, provides a clear-cut example of how pig models with naturally occurring mutations can be used to identify and characterize human disease-causing gene variants.

## RESULTS

### The autosomal-recessive inheritance mode of the disorder

**Fig. 1.**
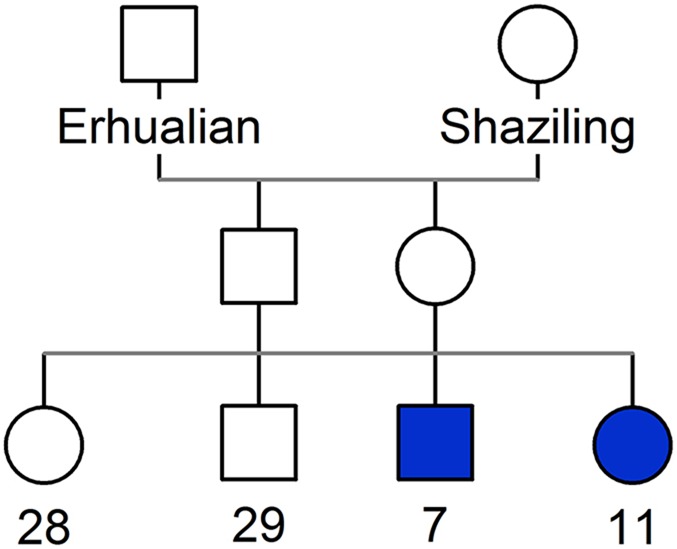
**The pedigree of the Erhualian × Shaziling F_2_ pig intercross segregating with autosomal-recessive microtia.** Squares indicate males and circles females. Blue symbols represent individuals with microtia. Clear symbols represent unaffected individuals. Numbers show the number of offspring with each phenotype.

From 2009 to 2014, we observed microtia-affected piglets in an Erhualian×Shaziling F_2_ family (Fig. 1). The inbred family was derived from a Chinese Erhualian founder boar and Shaziling founder sow. One F_1_ boar and one F_1_ sow were full-sib mated to produce 75 F_2_ offspring by six parities, of which 18 F_2_ individuals were affected with microtia. The disorder was inherited as a monogenic autosomal-recessive trait, as reflected by the expected 3:1 ratio of unaffected to affected animals and the appearance of the congenital anomaly in both males and females.

### Description of the phenotype in affected animals

**Fig. 2.**
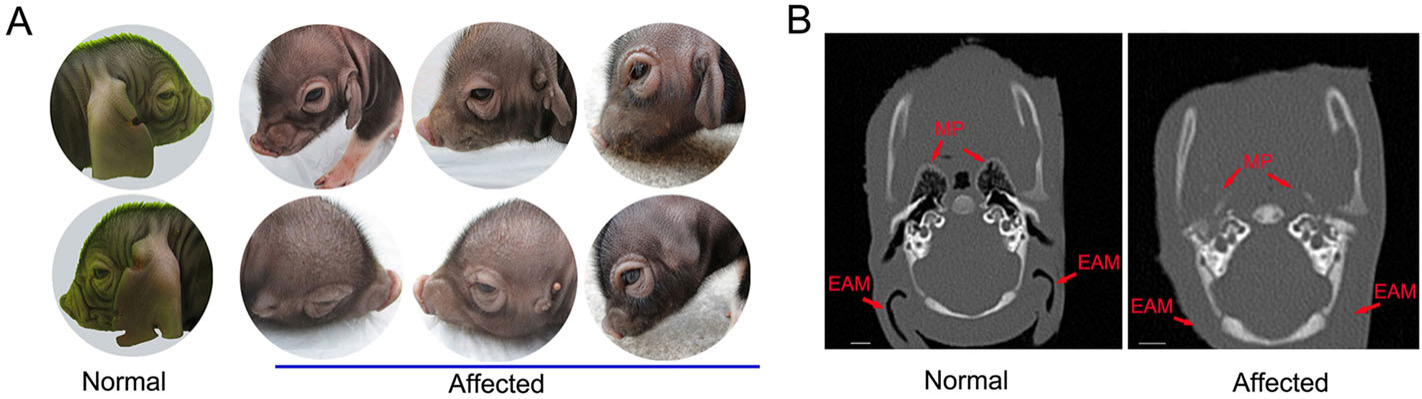
**Phenotypes of the normal and microtic ears in the Erhualian × Shaziling F_2_ pig intercross.** (A) Photographs of the normal and microtic ears in the F_2_ family. The external ears are short and severely narrowed, or even absent, bilaterally in the affected animals. (B) High-resolution CT imaging of the normal and microtic ears. In the affected pig, the inner-ear structure is normal; however, the external auditory meatus and mastoid process are absent. Scale bars: 5 mm. EAM, external auditory meatus; MP, mastoid process.

In the F_2_ family, all affected individuals were characterized by small, abnormally shaped, or absent, external ears on both sides (Fig. 2A). The abnormality was accompanied by severely narrowed, blocked or absent ear canals bilaterally, leading to hearing impairment. We performed a high-resolution computer tomography (CT) scan of the microtic ears. The CT images showed remarkably narrowed middle-ear cavities and nearly absent mastoid process, but normal inner-ear structures in affected animals (Fig. 2B). We noted that all affected animals showed normal oral and facial features but had eyelid defects, exhibited blunted response to external stimuli, had respiratory distress, lost suckling ability probably due to cleft palate, and usually died of malnutrition within 2 weeks after birth. No grossly visible defect was found in internal organs, including kidney, heart, lung and liver, after dissection of four affected piglets.

### Mapping of the responsible locus

**Fig. 3.**
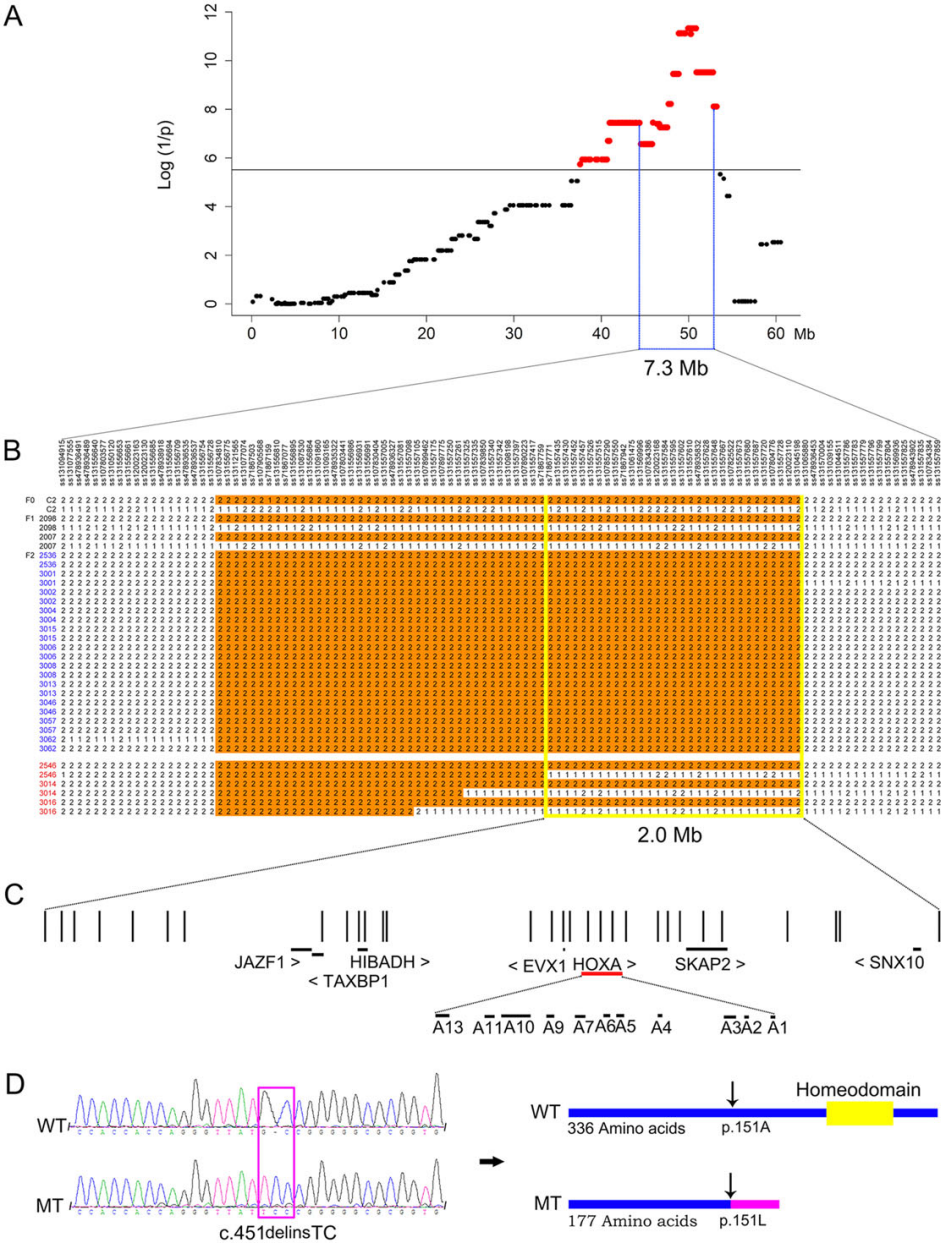
**Characterization of the causal mutation for the pig microtia.** (A) A genome-wide linkage analysis maps the disorder locus to pig chromosome 18. A strong association signal appears on this chromosome. SNPs surpassing the genome-wide significance threshold (black horizontal line) are highlighted in red. Negative log*P*-values are given in the *y*-axis. Genomic position of each SNP is shown in the *x*-axis. (B) Homozygosity mapping and recombination breakpoint analysis define the disorder locus within a critical region of ∼2.0 Mb. All affected F_2_ individuals, whose identities are indicated in blue, shared a homozygous interval of ∼5.0 Mb. Within the homozygous region, recombination events occur in three unaffected F_2_ individuals whose identities are denoted in red. The recombination breakpoints delineate the exact boundaries of the critical region from 48,877,373-50,901,463 bp (*Sscrofa* 10.2 assembly). (C) Significant SNPs and annotated genes in the 2-Mb critical region. Vertical lines represent 31 significant SNPs in the 60K chips. Horizontal lines represent annotated genes. A cluster of HOXA genes are located in this region. (D) Identification of the *HOXA1* c.451delinsTC polymorphism as the causal mutation. The left panel shows representative electropherograms for the *HOXA1* c.451delinsTC mutation from a wild-type (WT) and a homozygous mutant (MT) pig. The right panel illustrates that the frameshift mutation causes a truncated HOXA1 protein lacking the homeodomain.

To map the responsible locus, we genotyped 47 individuals, including two F_0_, two F_1_, 32 unaffected F_2_ and 11 affected F_2_ animals, from the 1-4 parities in the Erhualian×Shaziling F_2_ family using Illumina porcine 60K DNA chips. We explored 16,272 qualified SNPs to perform a genome-wide association analysis on the F_2_ family, after excluding the following single-nucleotide polymorphisms (SNPs): SNPs that had a low call rate (90%) or Hardy–Weinberg equilibrium deviation >10^−6^, were non-informative (minor allele frequencies <0.05), or were not mapped to the reference genome (*Sscrofa* 10.2). All significant SNPs were located on chromosome 18 (SSC18; supplementary material Fig. S1), and the strongest association signal appeared around an 890-kb region harboring 14 top SNPs with identical raw *P*-values of 4.71×10^−12^ (Fig. 3A). Next, we searched for an identical-by-descent (IBD) segment on SSC18 that was shared by affected F_2_ individuals. We found that all 11 affected F_2_ individuals were homozygous and shared identical alleles for 71 consecutive SNPs, including the top 14 SNPs, which defined a 5-Mb IBD haplotype (Fig. 3B). The disease-associated haplotype was inherited from the Shaziling founder sow. Subsequently, we examined recombination events in the IBD region on all F_2_ carriers (heterozygotes) with the normal ear phenotype. Three recombination events occurred in three F_2_ carriers. Of note, individual 2546 carried a non-recombinant disease-associated haplotype and a recombinant haplotype from both the Erhualian and Shaziling founders. The recombinant ss71867771–ss131045198 interval within the IBD region is a normal haplotype, which hence positioned the responsible locus downstream of ss71867771. Taken together, the IBD mapping and recombination breakpoint defined the responsible locus within an interval of ∼2.0 Mb delimited by markers ss71867771 and ss131045198 (Fig. 3B). The critical region harbors 17 annotated genes including a cluster of 11 HOXA genes in the *Sscrofa* 10.2 assembly (Fig. 3C).

### Identification of the causative mutation

We customized a capture array for a 2.5-Mb chromosomal segment encompassing the 2.0-Mb critical region. We then performed deep resequencing for the target region using genomic DNA of one affected F_2_ animal and its two F_1_ parents. We collected ∼2.3 million 2×90-bp paired-end reads in each animal and called more than 7446 variants corresponding to 84% of the region at ∼100× coverage depth (supplementary material Table S1). We noted that ∼16% of the region was not captured by the array. Nevertheless, all uncovered regions were intergenic or intronic. The strictly recessive mode of inheritance suggests a loss-of-function allele underlying the disorder. We thus focused on protein-altering variants in coding regions rather than regulatory mutations in non-coding regions. We then applied a series of exclusion steps to identify the causative mutation: (1) we removed variants that were heterozygous in the affected animal; (2) we excluded variants that were homozygous in one of the two parents; (3) we rejected variants that were inconsistent with the Mendelian inheritance model; (4) we excluded variants in the non-coding regions. Fifteen variants in five genes passed this series of exclusion criteria (Table 1). Next, we genotyped the 15 variants by Sanger sequencing (supplementary material Fig. S2) in a sample of 24 unrelated healthy boars from 11 Chinese diverse breeds. These animals are presumably non-carriers (wild-type homozygotes) because ear abnormalities have never been documented in the 11 breeds. All variants were segregating in the 24 individuals with relatively high frequencies of >0.1, except that *HOXA1* c.451delinsTC was fixed in these samples (supplementary material Table S2).

**Table 1.**
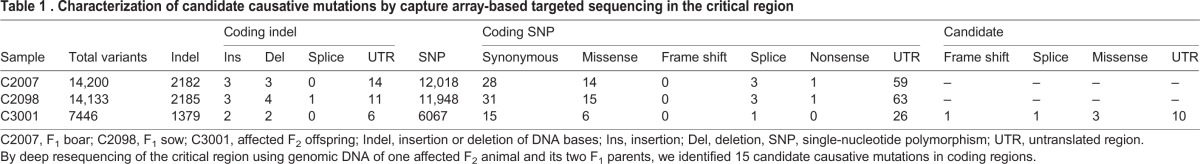
**Characterization of candidate causative mutations by capture array-based targeted sequencing in the critical region**

*HOXA1* c.451delinsTC occurs in exon 1 and causes a premature translation termination at residue 177 of HOXA1, resulting in a truncated protein. The truncated protein lacks a DNA-binding domain (homeodomain) that is essential for biological function of HOXA1 (Fig. 3D) (Alexander et al., 2009). *HOXA1* c.451delinsTC is thus a highly plausible loss-of-function variant. Truncating mutations in human *HOXA1* have been implicated in an autosomal-recessive microtia-associated syndrome characterized by disrupted inner ear, brainstem, cardiovascular and cognitive development. Three affected individuals also had external ear defects (Tischfield et al., 2005). *Hoxa1*-deficient mice present a disorganized middle ear, and underdeveloped or even absent inner ear (Chisaka et al., 1992; Lufkin et al., 1991). Given the implication of *HOXA1* in human and murine ear development, the *HOXA1* c.451delinsTC truncating mutation is thus a highly likely causative mutation for the external ear defect in pigs.

To obtain additional evidence for the causality of the truncating mutation, we further genotyped this mutation by *Sma*I PCR-RFLP (supplementary material Fig. S3) in a broad panel of 695 pigs from the F_2_ family and 32 diverse breeds. As expected for the causative variant, the *HOXA1* mutation cosegregated with the disease phenotype in the F_2_ family. All 18 affected animals were homozygous at this variation. Their parents, sibling carriers and the grandparent Shaziling carrier were heterozygous, and their unaffected siblings were homozygous (Table 2). Moreover, we found this variation exclusively in Shaziling pigs, with an allele frequency of 0.03, but not in 530 pigs from diverse other breeds (Table 2). This reinforces our assumption that *HOXA1* c.451delinsTC is the disease-causing mutation, and suggests that the microtia is a rare disease occurring solely in Shaziling pigs.

**Table 2.**
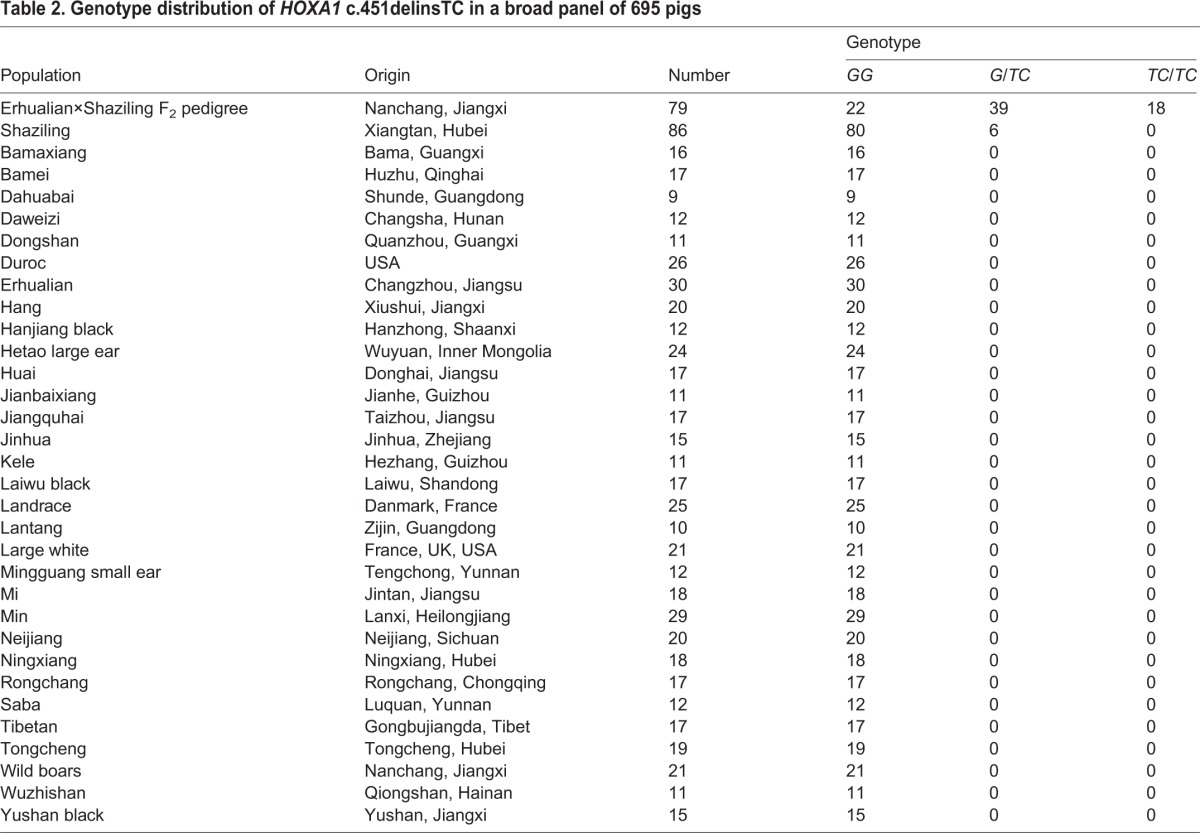
**Genotype distribution of *HOXA1* c.451delinsTC in a broad panel of 695 pigs**

### Detection of differentially expressed genes between affected and unaffected animals around the peak expression of *HOXA1*

To gain insights into the transcriptional network involving *HOXA1*, we performed RNA-Seq analysis on *HOXA1* wild-type and deficient pig embryos. Previous studies have shown that *HOXA1* is expressed in the anterior hindbrain of mouse and chicken embryos as early as E7.75, but expression recedes rapidly and is absent in this region by E9.0 (Makki and Capecchi, 2011). Owing to the very early and transient expression of this gene, we chose to harvest the pig embryos at the 9- to 12-somites stage (14.25 days after conception; supplementary material Fig. S4), corresponding to the E8.5 mouse embryo stage slightly after the peak expression of *HOXA1*. At this stage, the first and second branchial arches are present, whereas the third branchial arch is absent (supplementary material Fig. S4). It is known that the outer ear development is driven by the mesenchyme of the first and second branchial arches (Barrow et al., 2000; O'Gorman, 2005).

A total of 13 embryos were collected from a heterozygous F_2_ sow at 14.25 days after conception with a heterozygous boar. These embryos were genotyped for the *HOXA1* c.451delinsTC mutation site. For RNA-Seq analysis, two wild-type embryos and two homozygous mutant embryos were equally pooled to form the wild-type (*GG*) and mutant (*TC*/*TC*) samples, respectively. In total, we obtained over 52 million 2×90-bp paired-end clean reads from each of the two samples, corresponding to 4.7 Gb in size, of which ∼54% were mapped to the *Sscrofa* 10.2 assembly. Removal of reads that mapped to multiple locations in the assembly resulted in a ∼5.0% reduction in the total number of reads. Approximately 68% of clean reads mapped to 17,187 pig annotated genes, of which 10,896 genes had more than 80% gene coverage. We then compared expression files of these annotated genes between the two samples. Our ranking analysis (see Materials and Methods) yielded a list of 174 differentially expressed genes (DEGs) with a >1-fold change at a false discovery rate lower than 0.001.

Given that the assembly and annotation of the current pig reference genome are far from perfect, we further performed *de novo* assembly for ∼25-million unmapped reads. We generated 74,962 contigs at an average size of 351 bp in the mutant sample and 71,493 contigs at 355 bp in size in the wild-type sample. The overlapped contigs between the two samples corresponded to 51,432 Unigenes in the Swiss-Prot, KEGG, COG or NCBI NR protein database. We conducted a secondary differential analysis for these Unigenes and identified 170 DEGs. After removal of the overlapping genes between the 170 DEGs and the above-mentioned 174 DEGs, we obtained a final list of 337 DEGs, including 182 that were downregulated and 155 that were upregulated in the mutant (supplementary material Table S3). Two of the few known downstream targets of *HOXA1*, *ZIC1* and *FGFR3* (Makki and Capecchi, 2011, 2012) were in the list of 337 DEGs.

To test the robustness of the RNA-Seq data, we performed qPCR experiments on ten randomly selected DEGs using the same samples for RNA-Seq analysis. Of the ten genes, seven were validated by qPCR and three showed disconcordance in fold changes (supplementary material Table S4). Therefore, the qPCR results were generally in agreement with the RNA-Seq data.

To search for biological processes involving *HOXA1*, we conducted functional enrichment analysis for the 373 DEGs using the Ingenuity Pathway Analysis (IPA) tool (www.ingenuity.com/). The analysis identified a significant (*P*<0.05) overrepresentation of genes implicated in cancer, reproductive, neurological, skeletal and muscular, and renal and urological diseases (supplementary material Table S5). We also identified several molecular networks in which the DEGs were enriched. The top network was overrepresented by 25 DEGs, in which the top three functions are cardiovascular system development and function, cellular assembly and organization, and connective tissue development and function (Table 3 and supplementary material Fig. S5). The other overrepresented networks include top biological functions such as embryonic development, neurological disease, drug metabolism and molecular transport (Table 3). Moreover, we conducted the IPA-Tox analysis to assess molecular perturbation of the DEGs. The analysis generated ‘Acute renal failure panel’ (*P*=5.86E–04, ratio 6/62), ‘NRF2-mediated oxidative stress response’ (*P*=6.07E–04, ratio 12/234), ‘Renal necrosis/Cell death’ (*P*=8.13E–04, ratio 18/461), ‘Oxidative stress’ (*P*=2.62E–03, ratio 5/57) and ‘Renal safety biomarker panel’ (*P*=4.01E–03, ratio 2/6) as the top five most significant effects (supplementary material Table S5).

**Table 3.**
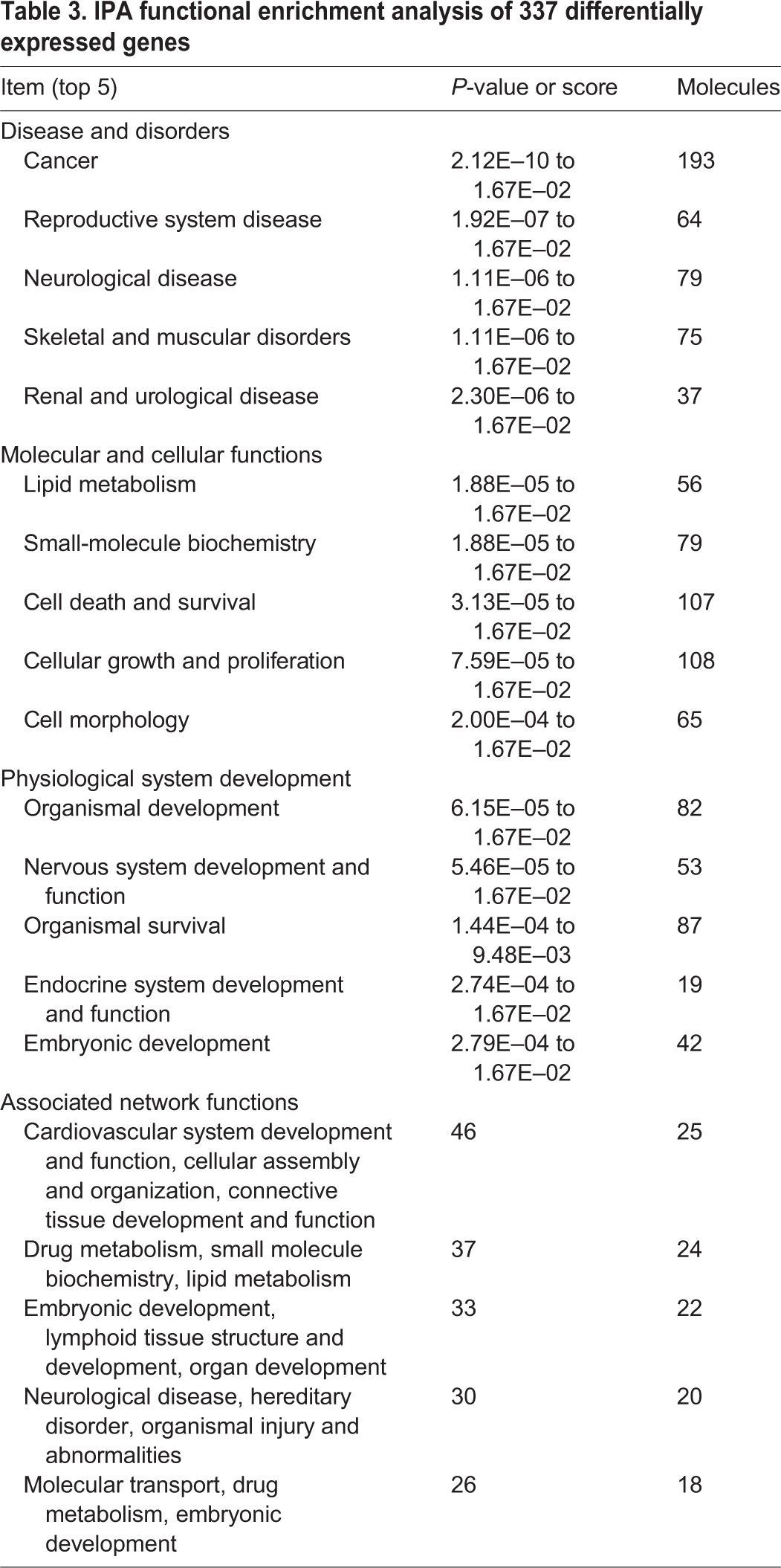
**IPA functional enrichment analysis of 337 differentially expressed genes**

### Characterization of candidate genes and causative mutations for human microtia

**Fig. 4.**
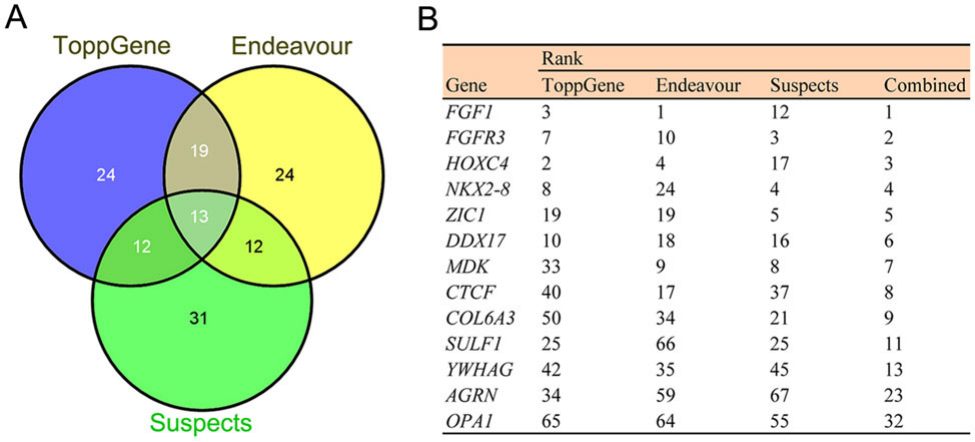
**Prioritization of candidate genes for human microtia syndromes by different disease-prediction algorithms.** (A) Venn diagram showing shared candidates among the top 20% by three candidate gene prioritization tools: ToppGene, Endeavour and Suspects. (B) A list of 13 candidate genes ranking in the top 20% in each of the three algorithms.

To characterize disease-causing candidate genes for human microtia, we performed computational prioritization analysis of the 337 DEGs. We composed a list of all the known genes that are associated with human microtia (supplementary material Table S6) and treated them as the training genes. We then characterized their signature similarity to the 337 DEGs by the combination of multiple data sources, including gene ontology, functional annotation, protein-protein interaction, gene expression, literature mining and sequence data. To increase the robustness of our analysis, we explored three commonly used methods in candidate gene prioritization: ToppGene (Chen et al., 2009), Endeavour (Tranchevent et al., 2008) and Suspects (Adie et al., 2006). Each of these methods generated a prioritized gene list (supplementary material Table S7). We searched for shared candidates among the top 20% ranked by the three prioritization tools, resulting in 13 consistently identified genes (Fig. 4). Of the 13 genes, *FGF1*, *FGFR3*, *HOXC4*, *NKX2-8* and *ZIC1* ranked as the top five candidates. This is supported by gene and protein interaction, motif sharing, annotation similarity and mouse phenotype data. The cross-algorithms prioritized genes, we believe, are strong candidates underlying human microtia.

**Fig. 5.**
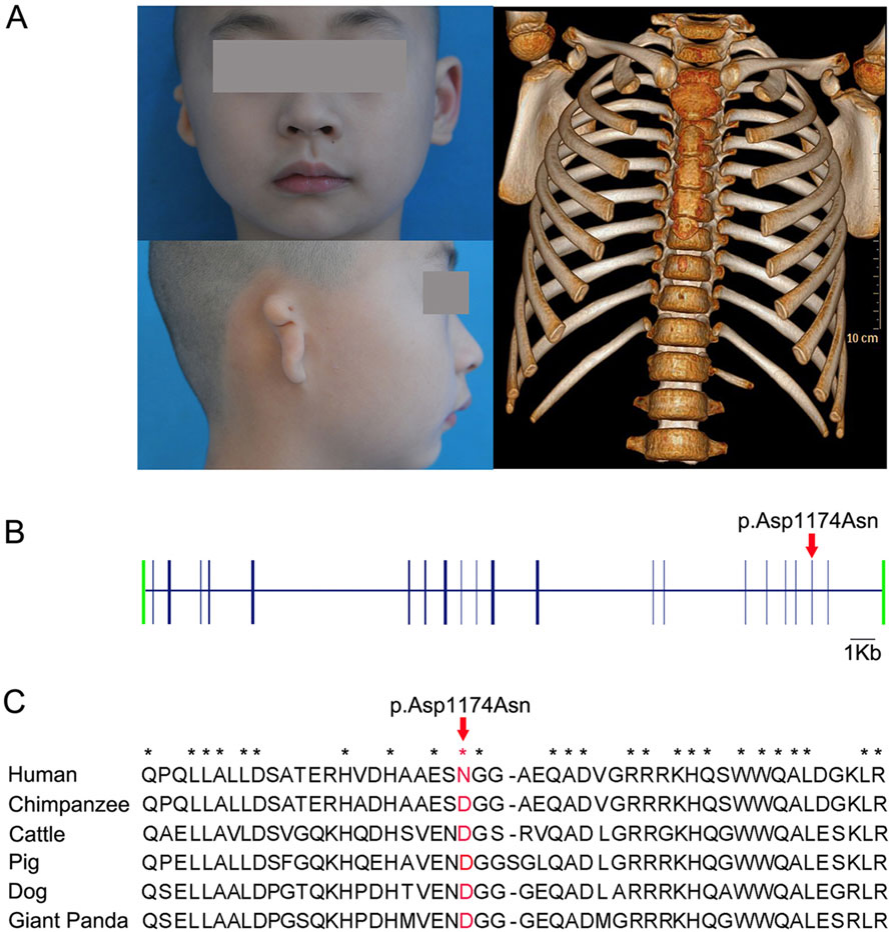
***EVC2* p.Asp1174Asn is a strong candidate causal mutation for human microtia.** (A) Phenotypes of an affected individual homozygous for the *EVC2* p.Asp1174Asn mutation. The patient has unilateral microtia and rib abnormality. (B) Schematic representation of the human *EVC2* gene and the location of the mutation. The open reading frame is indicated in blue and untranslated regions (UTRs) in green. (C) Multispecies alignment of the EVC2 protein sequence around the mutation (red). The amino acids that are labeled with a star are fully conserved in mammals.

To identify disease-predisposing variants in our highlighted candidate genes, we designed a set of primers (supplementary material Table S8) to amplify the coding regions and splice junctions of the top five prioritized genes together with *EVC2*, another functionally plausible gene (see Discussion). We detected 37 exonic variants by Sanger sequencing 54 amplicons in a sample of 147 humans with microtia. We then searched for potentially pathogenic variants by applying the following filtering criteria: we selected variants that have functional impacts as predicted by Polyphen2 (Adzhubei et al., 2013), are conserved across multiple species, and are absent in the database of The 1000 Genome project (http://www.1000genomes.org/), dbSNP (http://www.ncbi.nlm.nih.gov/SNP/) and The NHLBI Exome Variant Server (http://evs.gs.washington.edu/EVS/). Only one variant remained after filtering: *EVC2* p.Asp1174Asn (GenBank accession no. KM213243), an amino-acid change of aspartic acid to asparagine at position 1174 of human EVC2 (Fig. 5). The missense mutation is in a residue that is evolutionarily conserved across multiple mammals, including human, chimpanzee, cattle, pig, dog and panda, and is predicted to be ‘probably damaging’ at a probability score of 0.982 by PolyPhen2 (Adzhubei et al., 2013). The newly identified mutation occurred only in two sporadic patients with microtia and rib abnormalities. Both individuals were homozygous for the mutant allele. These findings led us to assume that *EVC2* p.Asp1174Asn is a candidate causative mutation for human microtia.

## DISCUSSION

In this study, we identified a truncating mutation (c.451delinsTC) of *HOXA1* as a candidate causative variant for congenital microtia in Chinese Shaziling pigs. Our genome-wide association mapping and recombination breakpoint analysis precisely localized the disorder-causing locus into a ∼2.0-Mb region. We then conducted capture array-based resequencing of the target region. We cautioned that our further analysis focused on protein-altering mutations in the region. Thus, we could not formally rule out the possibility that a synonymous or non-coding regulatory mutation in complete linkage disequilibrium with *HOXA1* c.451delinsTC is the actual causative variant. We might also have missed non-synonymous mutations in genes that are located in gap regions or are not annotated in the pig reference genome.

Although there are limitations of our analysis, we have obtained multiple lines of evidence that strongly support the causality of *HOXA1* c.451delinsTC in microtia. First, the strictly autosomal-recessive mode of inheritance suggests that a loss-of-function mutation is more likely responsible for the microtia that we investigated. Second, in the 2-Mb critical region, *HOXA1* c.451delinsTC is the only protein-altering mutation that occurs exclusively in Chinese Shaziling pig and the Erhualian×Shaziling F_2_ family. The mutation cosegregated with the microtia phenotype in the F_2_ family and was absent in 530 unaffected pigs from 31 diverse breeds. Third, *HOXA1* c.451delinsTC is a loss-of-function mutation that causes a truncated HOXA1 protein lacking the essential functional domain: the homeodomain. This domain is shared by all HOX genes and is required for the DNA-binding activities of HOX proteins functioning as transcription factors (Alexander et al., 2009). Fourth, the truncating mutation is actually within a functionally plausible candidate gene: *HOXA1*. *HOXA1* is a member of the HOX gene family, which plays a crucial role during embryonic patterning and organogenesis, specifically in the region of the developing hindbrain and branchial arches (Trainor and Krumlauf, 2001). It is known that the outer ear and middle ear originate from the mesenchyme at the first and second branchial arches (Barrow et al., 2000; O'Gorman, 2005). Microtia-affected piglets in our study manifest many symptoms that are present in humans with *HOXA1* truncating mutations (Bosley et al., 2008, 2007; Tischfield et al., 2005) or seen in *Hoxa1*-null mice (Chisaka et al., 1992; Lufkin et al., 1991), including abnormally shaped outer and middle ears, deafness, eyelid defects and, at least in affected humans, hypoventilation, swallowing dysfunction mainly due to cleft palate and mental retardation. Altogether, we assume that *HOXA1* c.451delinsTC causes the microtia and its associated abnormalities in Chinese Shaziling pigs. Our finding extends the phenotype and genotype of the homozygous *HOXA1* mutation clinical spectrum in mammals.

The *HOXA1*-deficient pigs presented here show a severity gradient of the external ear malformations, ranging from the relatively mild phenotype (visual appearance of the auricle) to the extreme anomaly (absent outer ears or anotia) (Fig. 2). The human Bosley-Salih-Alorainy (see OMIM reference no. 601536) and Athabascan brainstem dysgenesiss syndromes resulting from *HOXA1* loss-of-function mutations also have variable phenotypes among affected individuals, although horizontal gaze palsy and bilateral deafness have been considered the primary features (Bosley et al., 2008). The phenotypic variability implies that the *HOXA1* pathogenic mutations might not be universally penetrant in affected individuals. It is likely that other genes within or related to the HOX cascade partially compensate for dysfunction of the mutated *HOXA1* gene, leading to variable symptoms in affected individuals.

We note that the *HOXA1* mutation causes malformations of both the outer and middle ears in affected pigs, which is not surprising in view of their common embryological origin (Kountakis et al., 1995). In general, the better developed the outer ear, the better developed is the middle ear (Calzolari et al., 1999). The inner ear is derived from an epidermal otic placode at the level of the hindbrain (Nishikori et al., 1999), which is different from the origin of the outer and middle ear. Accordingly, most of the humans with microtia (Alasti and Van Camp, 2009) and the affected pigs in this study have a normal inner ear. Nevertheless, *Hoxa1-*null mice (Chisaka et al., 1992; Lufkin et al., 1991) and humans that are homozygous for *HOXA1* pathogenic variants (Bosley et al., 2008, 2007; Tischfield et al., 2005) sometimes show disrupted or even absent inner ears. The reason for the correlation between the external ear and inner ear remains unclear and needs further investigations.

Our RNA-Seq analysis provided a list of 337 genes that are differentially expressed between the early-somite-stage embryos of affected and unaffected animals. We assume that at least some of them are involved in the transcriptional network regulating the development of the outer ear. Our IPA analysis showed that these DEGs were enriched in functional categories related to cancer, reproductive, neurological, skeletal and muscular, and renal and urological disease. The IPA-Tox analysis indicated that molecular perturbation of the DEGs likely causes renal defects. Moreover, these DEGs were overrepresented in networks executing biological functions including embryonic development, neurological disease, drug metabolism and molecular transport. These findings are in agreement with the knowledge that *HOXA1* plays a crucial multifaceted role during embryonic patterning and organogenesis, and are also consistent with the fact that the human and mouse *HOXA1*-related phenotypic presentations often have cardiovascular defects, renal abnormalities, rib deformities and mental retardation (Barrow et al., 2000; Chisaka et al., 1992; Harris et al., 1996; Mastroiacovo et al., 1995; Wang et al., 2001). Although we did not observe apparent malformations of heart and kidney in *HOXA1*-deficient pigs by visual inspection, we speculate that the affected pigs might have cardiac and renal defects. Further in-depth clinical investigations would need to be conducted to testify our hypothesis.

Accumulating lines of evidence have shown that closely related disease-influencing genes are most likely to have similar molecular signatures, including similar expression profiles and protein domains, participation in the same signaling or metabolic pathways, and phenotypically similar abnormities in knockout mice (Feldman et al., 2008; Goh et al., 2007). Here, we used three computational algorithms (ToppGene, Endeavour and Suspects) to prioritize gene lists from the 337 DEGs by searching for their similarities to the 32 previously known microtia-implicated genes. We found that 13 genes ranked in the top 20% across the three algorithms. These genes are thus appealing candidates for human microtia-syndromes and will be the basis of future investigations.

We are particularly interested in the top five prioritized genes that are highly plausible candidates. *FGF1* and *FGFR3*, ranking in the top list, are involved in the FGF signaling pathway, which plays a crucial role in pinna development (Abu-Issa et al., 2002; Wright and Mansour, 2003). Mice deficient for paralogs of the two genes, including *FGF3*, *FGF8* and *FGFR1*, exhibit small outer ears (Abu-Issa et al., 2002; Partanen et al., 1998; Wright and Mansour, 2003). *HOXC4*, one of the HOX genes, has been associated with the VACTERL association disease characterized by vertebral abnormities, anal atresia, cardiac defects, tracheal anomalies, esophageal atresia and renal abnormalities (Genecards database, http://www.genecards.org/). Some individuals with the disease also show external ear malformations that resemble the microtia syndromes (Solomon, 2011). *NKX2-8* is also a homeobox gene that has a potential role in the development of a variety of tissues (Apergis et al., 1998; Lin et al., 2013; Safra et al., 2013). Recently, *NKX2-8* variants have been implicated in neural tube defects in dogs and humans (Safra et al., 2013). The defects include neurological impairment, vertebral deformities, brain malformations, and genitourinary and gastrointestinal disorders (Copp et al., 2013), phenotypes that sometimes co-present with microtia. Mutations in *NKX5-3* (also known as *HMX1*), an important paralog of *NKX2-8*, causes an oculo-auricular syndrome characterized by abnormalities of the external ear and the eye (Schorderet et al., 2008). *ZIC1* encodes a member of the ZIC family of C2H2-type zinc-finger proteins. This gene is known to be expressed in the posterior hindbrain from which cardiac neural crest cells arise. It has been reported that *HOXA1* likely acts upstream of *ZIC1* in the regulation of neural crest specification (Makki and Capecchi, 2011, 2012). Downregulation of *ZIC1* could be the reason for the outflow tract defects in humans and mice homozygous for *HOXA1* mutations (Makki and Capecchi, 2011, 2012). Although we did not identify disease-predisposing variants within the five genes in a sample of 147 affected humans, further investigations on larger patient cohorts could yield causative mutations for the genetic disorder.

In humans, around 20-60% of individuals with microtia have additional anomalies (Mastroiacovo et al., 1995; Shaw et al., 2004). The most commonly associated syndromes include limb-reduction defects, polydactyly, vertebral abnormities, cardiac defects, renal anomalies and cleft lip or palate (Harris et al., 1996; Mastroiacovo et al., 1995; Wang et al., 2001). One explanation for the association is that genes that are essential for the development of the external ear, such as *HOXA1*, could also be involved in the development of other tissues or organs. Pathogenic variants within these genes might simultaneously cause the external ear malformations and other anomalies. Here, we identified 337 DEGs that are likely targets of *HOXA1*. Therefore, mutations in these genes could result in microtia and other malformations. Given that the top ranked genes were sequenced and found not to harbor any putative coding variants of interest, we moved on to extend our gene list to include those DEGs that have been implicated in abnormalities that are also found in microtia-associated syndromes. *EVC2* stands out as an interesting candidate for the microtia syndrome. *EVC2* encodes a protein that plays a crucial role in bone formation and skeletal development. Mutations in this gene cause Ellis van Creveld syndrome and Weyers acrofacial dysostosis, clinically characterized by polydactyly, short ribs and limbs, growth retardation, congenital heart disease and ectodermal dysplasia (Baujat and Le Merrer, 2007; D'Asdia et al., 2013; Shen et al., 2011; Ye et al., 2006), which are phenotypes commonly associated with microtia. Here, we identified a nonsynonymous mutation in exon 20 of the *EVC2* gene that is predicted to result in substitution of Asp1174 with Asn. The mutation alters an evolutionarily conserved amino acid residue, presumably leading to functional consequences as predicted by PolyPhen2. Intriguingly, the mutation is a previously unreported variant that appeared only in two unrelated patients with microtia and rib abnormalities. These findings support the involvement of the *EVC2* mutation in microtia-associated malformations, although formal proofs needs to come from the identification of additional patients with the mutation and further functional validation of the effect of the mutation on the EVC2 protein. To our knowledge, this is the first report of microtia associated with the *EVC2* mutation. Hence, our data further extend the spectrum of malformation syndromes due to *EVC2* mutations. Interestingly, a susceptibility locus for isolated bilateral microtia in a five-generation Chinese pedigree has been recently mapped to a 10-Mb region encompassing the *EVC2* gene (Li et al., 2014). Our results warrant further investigation of the *EVC2* mutations for the microtia phenotype in the pedigree.

## MATERIALS AND METHODS

### Ethics statement

All procedures involving animals are in compliance with the care and use guidelines of experimental animals established by the Ministry of Agriculture of China. The Ethics Committee of Jiangxi Agricultural University specifically approved this study.

Human patients with microtia (*n*=147) were diagnosed at the Plastic Surgery Hospital, Peking Union Medical College of China. Informed consent was obtained from each donor's parents or guardians. The clinical investigation and sample collection protocol conforms to the ethical guidelines of the Declaration of Helsinki and was approved by the Ethics Committee of the Plastic Surgery Hospital, Peking Union Medical College.

### Pig sample selection

We ascertained an F_2_ inbreeding family, derived from a cross between one Erhualian boar and one Shaziling sow, that was segregating for an autosomal-recessive form of bilateral microtia. The F_2_ population resulting from the full-sib mating of one F_1_ male and one F_1_ female includes 18 affected individuals and 57 unaffected individuals (Fig. 1). Veterinary evaluation of congenital abnormalities was conducted on all of the affected individuals. A high-resolution CT imaging was further performed on these animals to detect anomalies of the middle and inner ears. All pigs were raised under a consistent indoor condition and were fed *ad libitum* with a diet containing 16% crude protein, 3100 kJ digestible energy and 0.78% lysine in the experimental farm of Jiangxi Agricultural University (China).

### Genome-wide association study and fine mapping

Genomic DNA of each animal was extracted from ear or tail tissues using a standard phenol/chloroform method. A total of 47 pigs from the Erhualian×Shaziling F_2_ family were genotyped using the Porcine SNP 60K BeadChips (Illumina) according to the manufacturer's protocol. Genomic position of each SNP corresponds to the *Sscrofa* 10.2 assembly (Groenen et al., 2012). Quality control of the individuals and autosomal SNPs were performed after checking if the genotyping failure was associated with the phenotype by PLINK command option ‘test-missing’. Qualified SNPs were filtered by excluding those with call rates <90%, minor allele frequencies <0.05, Hardy–Weinberg equilibrium deviation >10^−6^ or unknown genomic position. A genome-wide association study was then conducted using the qualified SNPs and a haplotype-based model as described previously (Druet and Georges, 2010). Briefly, haplotype of each chromosome was reconstructed with the DualPHASE software (Druet and Georges, 2010) for all of the 47 pigs in the F_2_ family. Then, the phenotypic difference between F_2_ individuals of different founder origins at each SNP site was evaluated by the *t*-test. The genome-wide significant threshold was determined by Bonferroni correction. It was defined as 0.05/*N*, where *N* is the number of informative SNPs. To fine map the disease-causing locus, we searched for the homozygous segment shared by all affected F_2_ individuals along chromosome 18 by visual inspection. Recombination breakpoint analysis was conducted to define the boundary sites of the critical region as reported in our previous study (Ren et al., 2012).

### Array-based capture and resequencing

Customized probes were designed to hybridize with DNA sequences within a 2.5 Mb genomic region harboring the disorder-predisposing locus on chromosome 18. Array-based capture was conducted on the NimbleGen SeqCap EZ Designs Library Capture platform (Roche). Enriched hybridization libraries with ∼300 bp insert size were then sequenced on a HiSeq 2000 sequencer (Illumina) to generate paired-end reads (2×90 bp) at an approximate 100× coverage. The raw sequencing data was qualified by removing adapter sequences, fuzzy base *N*, bases with quality scores <20 and reads shorter than 20 bp. Variants were called from the qualified clean data using the bioinformatics pipeline developed by BGI (Shenzhen). Briefly, filtered reads from all individuals were aligned to the pig reference genome (*Sscrofa* 10.2 assembly) by BWA (Li and Durbin, 2009). SOAPsnp (Li et al., 2009b) was then used for SNP calling for each sample. Before SOAPsnp SNP calling, SAMTOOLS (Li et al., 2009a) was used for sorting, merging and removal of potential PCR duplications. SNPs with depth less than 6 or more than 70 or with distance less than 5 bp to their former SNPs were removed. A high-quality site should fit the criteria that the SNPs of all samples have a SOAPsnp quality score of no less than 20. Resequencing data from this study are available in Sequence Read Archive (SRR1521087, SRR1521088 and SRR1521089).

### Sanger sequencing

We used Sanger sequencing to genotype selected variants. Primers (supplementary material Table S8) were designed for 15 candidate causative mutations. Genomic DNA of 24 representative individuals was amplified in a routine way at optimal annealing temperatures. PCR products were directly sequenced on a 3130XL capillary sequencer (Applied Biosystem). The Sanger sequence data were analyzed with Sequencer 5.1 (Genecode).

### *Sma*I PCR-RFLP

*HOXA1* c.451delinsTC was genotyped by *Sma*I PCR-RFLP. Genomic DNA was amplified in a volume of 15 μl with forward (5′-TGGACA-ATGCAAGAATGAGC-3′) and reverse (5′-CCCACGTCCTACTTCCA-AAA-3′) primers. PCR products were digested with 5 units of the restriction enzyme *Sma*I (Takara) at 37°C for 6 h. The restriction fragments were then electrophoresed in 1.5% agarose gels and visualized on an ultraviolent chamber (Syngene). The wild-type allele (*G*) was represented by a restriction fragment of 891 bp and the mutant allele (*TC*) by two fragments of 438 bp and 453 bp.

### RNA sequencing

To collect *HOXA1*-null and wild-type embryos at the 9- to 12-somites stage, we slaughtered a heterozygous F_2_ sow at day 14.25 after mating with an F_2_ boar that was heterozygous at the *HOXA1* c.451delinsTC mutation site. Embryos were harvested from the uterus with PBS containing 1% BSA (Ambion). Each embryo and its extraembryonic tissue were isolated into a separate dish filed with cold PBS under a microscope. Extraembryonic tissues were removed for DNA extraction and genotyping of *HOXA1* c.451delinsTC by *Sma*I PCR-RFLP, and the number of somites was counted. Each embryo was then transferred into a 1.5 ml tube filled with 100 μl lysate of the MICROBEnrich Kit (Ambion), and stored in liquid nitrogen for subsequent RNA extraction. Total RNA was isolated from each embryo using Dounce Tissue Grinders (Shenggong) and the MICROBEnrich Kit (Ambion). The concentration and quality of RNA was measured by a 2100 bioanalyzer (Agilent). RNA samples were qualified to have RNA Integrity Numbers of 9.8-10 and concentrations of more than 100 ng/μl. Two wild-type and two mutant embryos at the 9- to 12-somites stage were equally pooled to form two RNA samples for subsequent RNA-Seq experiments.

cDNA libraries were constructed from the two RNA samples using the TruSeq RNA Sample Preparation Kit (Illumina). Pair-end reads (2×90 bp) were then generated on a HiSeq 2000 sequencer (Illumina). Next, trimmed clean reads were mapped to the pig reference genome (*Sscrofa* 10.2) allowing five mismatches using the SOAP2 software (Li et al., 2009c). Gene expression levels were further analyzed to identify DEGs between the wild-type and mutant RNA samples. RPKM (reads per kilobase transcriptome per million mapped reads) was used as a measure of gene expression (Mortazavi et al., 2008). The proportion of read counts for each gene in relation to the total read counts in each sample was determined according to the following formula:

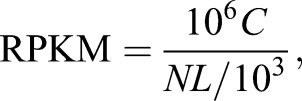


whereby, *C* means the number of reads that are uniquely aligned to gene A, *N* means the total number of reads that are uniquely aligned to all genes and *L* means the number of bases on gene A. Fold change was determined as the ratio of RPKM of the wild-type sample versus RPKM of the mutant sample. Then, we adopted a strict algorithm to identify DEGs between the wild-type and mutant samples. The probability that gene A was expressed equally between the two samples was calculated using the following equations:

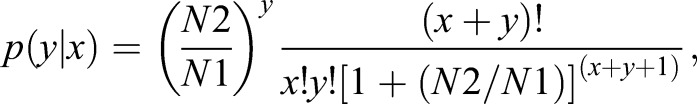


where *N*1 and *N*2 are the total number of unambiguous clean reads aligned to all genes in the wild-type and mutant samples, respectively; *x* and *y* are the number of unambiguous clean reads corresponding to gene A in the wild-type and mutant samples, respectively (Audic and Claverie, 1997). This probability followed a Poisson distribution. Next, the false discovery rate (FDR) method (Benjamini and Yekutieli, 2001) was used to correct for false-positive (type I) and false-negative (type II) errors resulting from multiple tests. Finally, we used an FDR of no more than 0.001 (FDR≤0.001) and the difference ratio of PRKM between the wild-type and mutant samples no less than 1 (Log2Ratio≥1) as the significant threshold to identify DEGs.

As to the unmapped clean reads, we carried out *de novo* assembling with Trinity (Grabherr et al., 2011). The resulting read contigs (Unigenes) were then compared to protein databases including NR, Swiss-Prot, KEGG and COG using the BLASTx algorithm (e-value<0.00001) to identify human homologous genes. DGEs were again defined under the same criteria for the mapped clean reads. DEGs of these two sources were merged to form a final list of genes that were differentially expressed between the two samples. Enrichment analyses of biofunctions and networks were conducted on the DEGs by the use of IPA (Ingenuity Systems, www.ingenuity.com/). The IPA assigned the top three biological functions for each network it identified. A significance level of corrected *P*-values less than 0.05 was used for all IPA tests. RNA-Seq data from this study are available in Sequence Read Archive (SRR1519321 and SRR1519322).

### Real-time quantitative PCR

The two RNA samples for RNA-Seq were also used to validate ten randomly selected DEGs by real-time quantitative PCR with *RPL19* as the reference gene. Gene-specific primers (supplementary material Table S8) were designed by AlleleID 6.0 (Applied Biosystems). Real-time PCR reactions were performed using 1× Power SYBR Green PCR Master Mix on a 7500FAST thermal cycler (Applied Biosystem) in four replicates. The annealing temperature was 60°C and the data-collection temperature was set to 75°C to avoid signal collection of possible primer dimers. Relative expression levels of the target genes were calculated with the 2^−ΔΔCt^ method (Schmittgen et al., 2008), normalizing to the reference gene *RPL19*. Data were expressed as mean fold change relative to wild type. Unpaired, two-tailed Student's *t*-test was used to calculate *P*-values between the wild-type and mutant samples.

### Candidate gene prioritization

We used three online algorithms comprising Suspects (Adie et al., 2006), ToppGene (Chen et al., 2009) and Endeavour (Tranchevent et al., 2008) to prioritize the identified 337 DEGs. A list of 32 genes that are well-known to be associated with external ear malformation in human and mouse were used as the training genes. We picked up the top 20% ranking genes to identify the overlapping ones across the three algorithms.

### Sequencing of human samples

Primers (supplementary material Table S8) were designed to amplify exonic regions of the target genes. Purified PCR products were bi-directionally sequenced on a 3130XL capillary sequencer (Applied Biosystems). Sequences were aligned by the Sequencer 5.1 (Genecode) to identify variants. The presence of variants was tested by searching against the database of the 1000 Genome Project (http://www.1000genomes.org/) and the NHLBI Exome Variant Server (http://evs.gs.washington.edu/EVS/U). Functional significance of the identified variants was evaluated by Polyphen2 (Adzhubei et al., 2013).

## Supplementary Material

Supplementary Material
