# Supplementary material for: Understanding the molecular mechanisms of human microtia via a pig model of HOXA1 syndrome
Source: Dis Model Mech. 2015 Jun 1;8(6):611–22. doi: 10.1242/dmm.018291 (PMC4457031; doi:10.1242/dmm.018291)
Supplement: Supplementary Material [file supp_8_6_611__index.html]

Understanding the molecular mechanisms of human microtia via a pig model of HOXA1 syndrome — Supplementary Material 

# Understanding the molecular mechanisms of human microtia via a pig model of *HOXA1* syndrome

## DMM018291 Supplementary Material

**Files in this Data Supplement:**

- **Supplementary Material**
