# Supplementary material for: Understanding the molecular mechanisms of human microtia via a pig model of HOXA1 syndrome
Source: Dis Model Mech. 2015 Jun 1;8(6):611–22. doi: 10.1242/dmm.018291 (PMC4457031; doi:10.1242/dmm.018291)
Supplement: Supplementary Material [file supp_8.6.611_DMM018291.pdf]

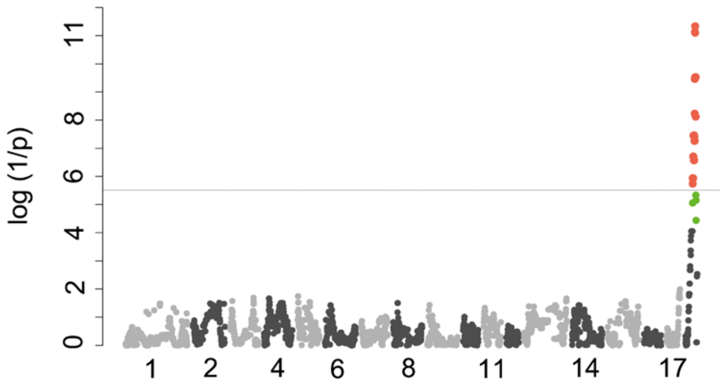

Figure S1 Manhattan plots for the genome-wide association analysis of the responsible locus. In the plots, negative $\log_{10}$ *P*-values of the qualified SNPs were plotted against their genomic positions. The solid horizontal line indicates the genome-wide significant threshold.

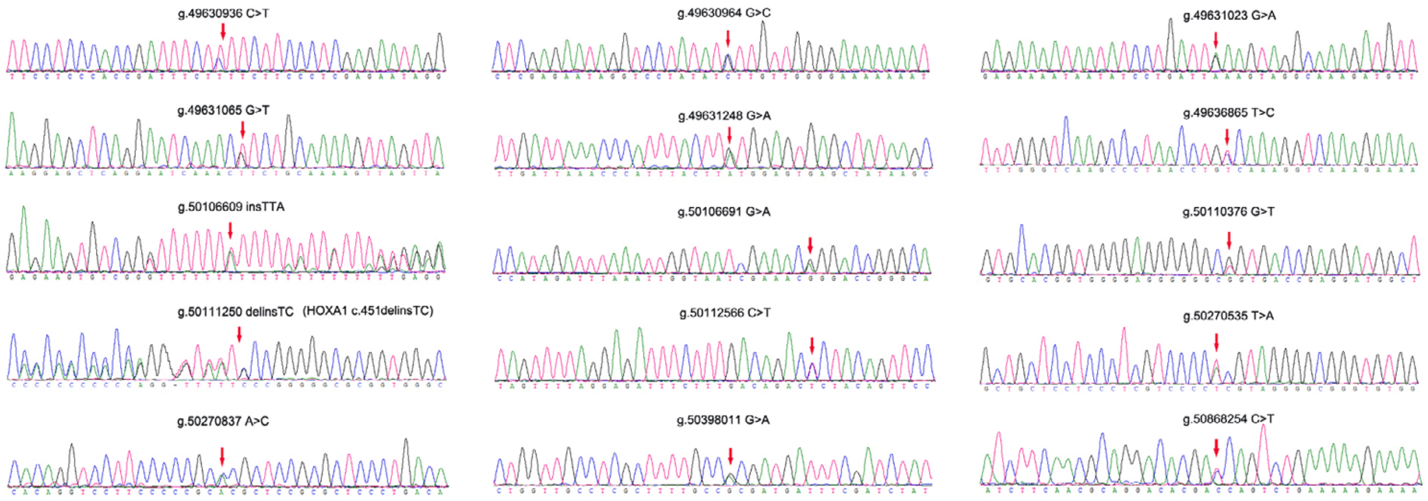

Figure S2 Representative sequence electropherograms for 15 candidate causative mutations.

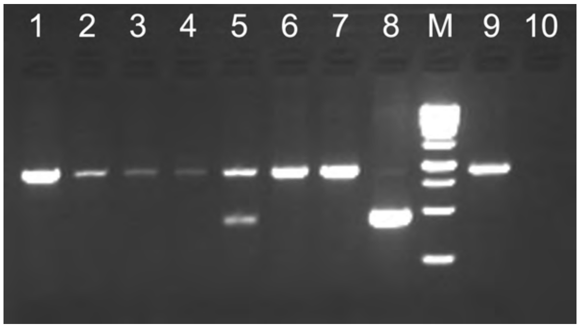

Figure S3 Genotype patterns at the *HOXA1* c.451delinsTC mutation site by a *Sma* I PCR-RFLP assay. Lines 1-4, 6-7 and 9: *G*/*G*. Line 5: *G*/*TC*. Line 8: *TC*/*TC*. Line 11: H_2O. M: 1 Kb marker.

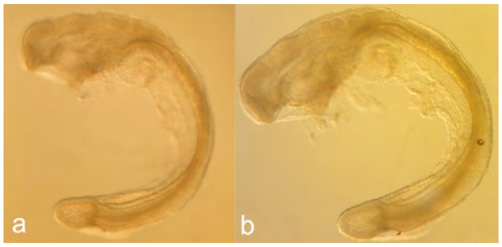

Figure S4 Microscopic imaging of a 14.25-day embryo. (A) Unaffected individual. (B) Affected individual.

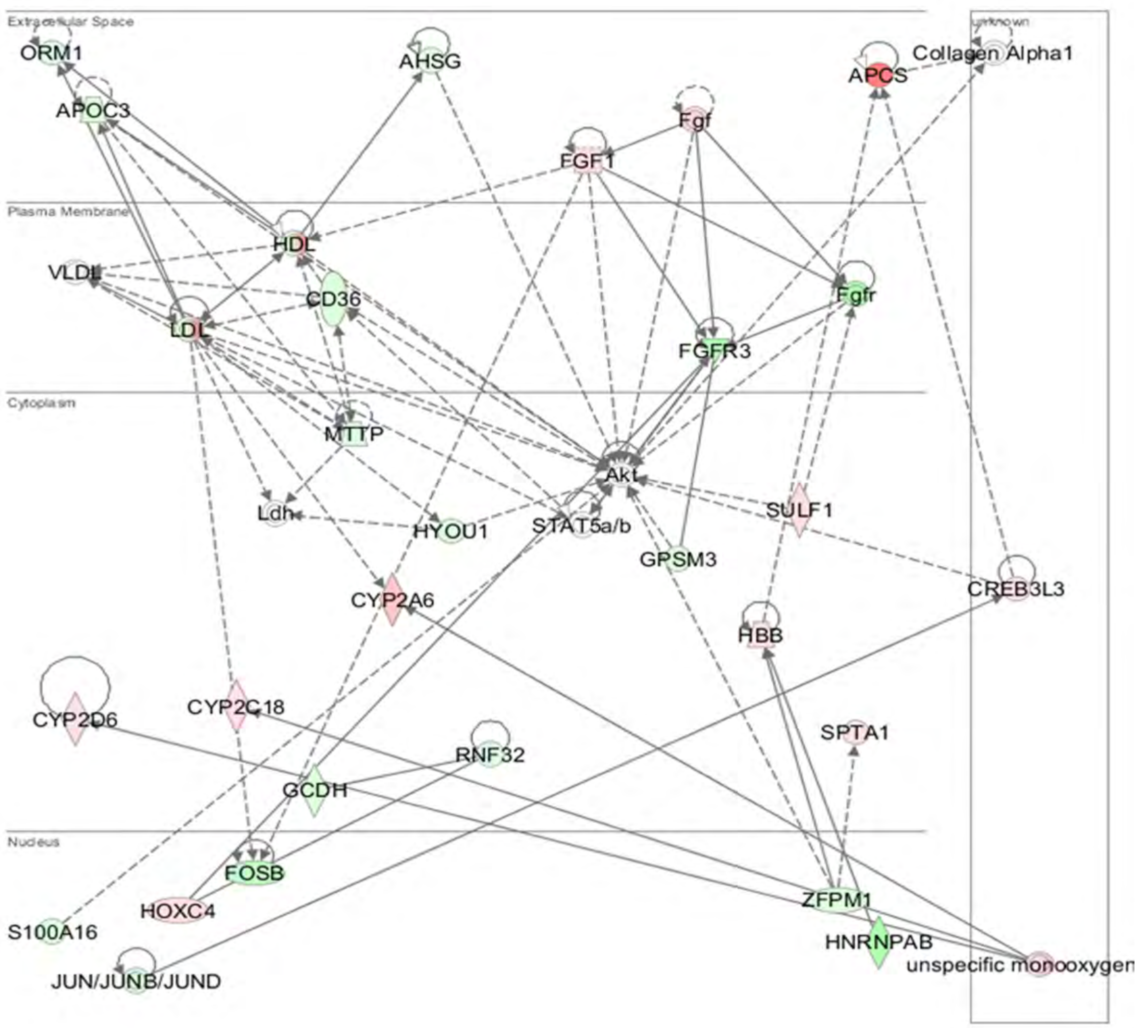

Figure S5 The top network overrepresented by 25 differentially expressed genes. The functions of this network include "cardiovascular system development and function", "cellular assembly and organization" and "connective tissue development and function". Nodes in red indicate up-regulation and nodes in green indicate down-regulation.

Table S1 A description of sequencing data in the target region

Download Table S1

Table S2 Genotype distribution of 15 candidate causative mutations in 24 Chinese indigenous pigs.

Download Table S2

Table S3 A full list of 337 differentially expressed genes between 14.25-day embryos of affected and unaffected individuals.

Download Table S3

Table S4 Validation of RNA-Seq results by quantitative PCR on 10 differentially expressed genes.

Download Table S4

Table S5 Analysis of IPA enriched tox functions and lists for 337 differentially expressed genes.

Download Table S5

Table S6 A list of 32 genes that are known to be associated with human or mouse microtia.

Download Table S6

Table S7 The prioritized gene lists resulting from three computational algorithms including ToppGene, Endeavour and Suspects.

Download Table S7

Table S8 Primers used in this study.

Download Table S8
